# Determination of Exogenous Adrenaline Levels in Patients Undergoing Cardiopulmonary Resuscitation

**DOI:** 10.1021/acsomega.3c00555

**Published:** 2023-05-25

**Authors:** Mehmet Altuntaş, Derya Bal Altuntaş, Sema Aslan, Ersin Yılmaz, Ercan Nalbant

**Affiliations:** † Faculty of Medicine, Department of Emergency Medicine, Recep Tayyip Erdogan University, Rize 53100, Turkey; ‡ Faculty of Engineering and Architecture, Department of Bioengineering, Recep Tayyip Erdogan University, Rize 53100, Turkey; § Department of Chemistry, Faculty of Science, Muğla Sıtkı Koçman University, Muğla 48170, Turkey; ∥ Department of Statistics, Muğla Sıtkı Koçman University, Muğla 48170, Turkey

## Abstract

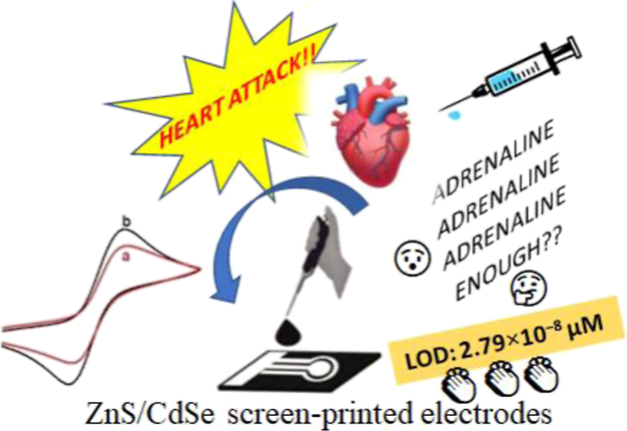

Core–shell quantum dot ZnS/CdSe screen-printed electrodes were used to electrochemically measure human blood plasma levels of exogenous adrenaline administered to cardiac arrest patients. The electrochemical behavior of adrenaline on the modified electrode surface was investigated using differential pulse voltammetry (DPV), cyclic voltammetry, and electrochemical impedance spectroscopy (EIS). Under optimal conditions, the linear working ranges of the modified electrode were 0.001–3 μM (DPV) and 0.001–300 μM (EIS). The best limit of detection for this concentration range was 2.79 × 10^–8^ μM (DPV). The modified electrodes showed good reproducibility, stability, and sensitivity and successfully detected adrenaline levels.

## Introduction

Commercial forms of catecholamines have been developed for the treatment of many different diseases. Adrenaline (epinephrine), a catecholamine that can be administered as a drug, is most commonly used in emergency medicine settings (e.g., for cardiac arrest, anaphylaxis, and septic shock). It is the primary drug administered to reverse cardiac arrest during cardiopulmonary resuscitation (CPR).^1^ The administration of 1 mg of adrenaline every 3–5 min to a patient with cardiac arrest is recommended.^2^ Adrenaline is a sympathomimetic drug; it increases the flow of blood and oxygen to the heart during CPR by increasing the aortic diastolic pressure and coronary perfusion pressure. It also stimulates spontaneous heart contractions, and it increases the chance of success of defibrillation by making ventricular fibrillation have large fluctuations and increases the heart rate, blood pressure, and oxygen demand of the heart muscle. When injected intravenously, adrenaline is rapidly depleted from the circulatory system. When administered subcutaneously or intramuscularly, it exhibits a rapid onset and a short duration of action.^3^

The determination of adrenaline concentrations in pharmaceutical samples and various biological fluids, such as plasma and urine, is important for pharmacological research and nerve physiology and life science studies.^4^ Since the concentrations of catecholamines in biological fluids are low, precise analysis methods are required. High-performance liquid chromatography, spectrophotometry, fluorimetry, capillary electrophoresis, chemiluminescence, and electrogenerated chemiluminescence are some methods of analysis that have been used to determine adrenaline concentrations in previous studies.^5−8^ However, since adrenaline molecules are easily oxidized, adrenaline levels have frequently been determined using electrochemical methods in recent studies.^9^ Additionally, electrochemical methods of determination are among the most attractive and convenient methods since they are simple and quick, do not require any preliminary preparation, and do not require expensive equipment. When high precision is not required, using bare electrodes in electroanalytical studies offers some advantages; the use of a bare electrode is a low-cost, time-saving, simple, and sustainable procedure. On the other hand, since adrenaline has a slow rate of electron transfer and adsorbs onto the electrode surface, the electrochemical reaction of adrenaline on the surface of bare electrodes is weak.^10^ Modifying the bare electrode surface with various materials is effective for overcoming these limitations. Among these substances, polymerizable molecules are the most commonly used.^11^

Quantum dots offer a very high electrochemical contribution to the sensors in experimental and practical terms. Especially, core–shell quantum dot structures including transition metals provide superior current responses due to their high band gaps. Some examples of core–shell quantum dot structures used in sensors reported earlier include CdS, CdSe, ZnS, and CdS combinations.^12,13^ In this study, for the first time, the adrenaline level in the blood of patients having a heart attack was determined electrochemically using CSQD-ZnS/CdSe quantum dots.

Adrenaline is the primary drug of choice for resuscitation, and it increases the likelihood of return of spontaneous circulation (ROSC) after cardiac arrest. However, the long-term consequences of its use remain unclear. A few animal studies have shown that although adrenaline increases the blood flow to vital organs in general, it may worsen microcirculation. While a large number of clinical, observational studies have reported correlations between adrenaline injection and worse long-term consequences, some observational studies have shown correlations between early adrenaline injection and better long-term consequences. In conclusion, there is still no clarity regarding the role of adrenaline injection in patients with cardiac arrest.^1^

The aim of this study was to develop an electrode capable of detecting exogenous adrenaline levels with high sensitivity in patients undergoing CPR.

## Results and Discussion

This study evaluated CSQD-ZnS/CdSe SPEs as a potential electrochemical method for determining adrenaline levels in patients undergoing CPR.

### Morphological Characteristics

**Figure 1:**
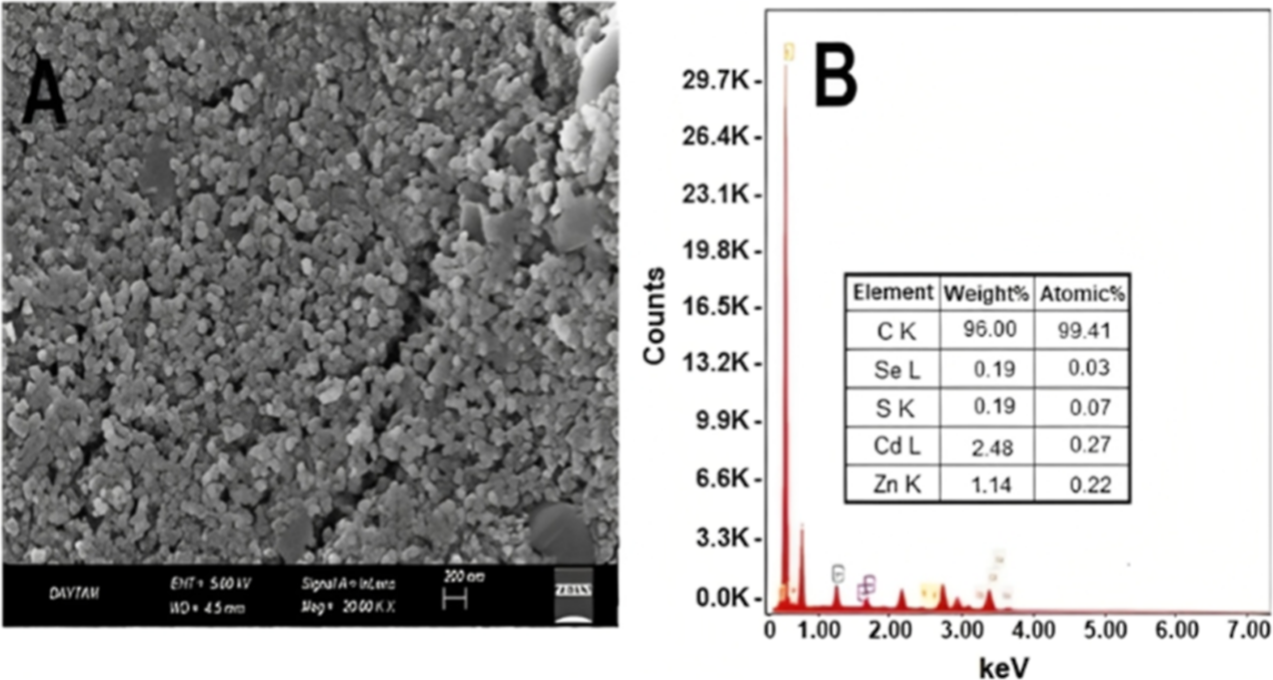
(A) Scanning electron microscopy image of the CSQD-ZnS/CdSe SPE. (B) EDS spectrum of the electrode showing the constituent percentages.

Prior to the sensor measurements, the surface and the composition of the electrode were investigated. The morphology and microstructure of the CSQD-ZnS/CdSe SPE were analyzed using scanning electron microscopy and energy-dispersive X-ray spectroscopy (EDS). Quantum dots can be seen in Figure 1A.

The constituents of the hybrid quantum dot structure are also shown in the inset in Figure 1B. The electrode showed a unique morphology that enabled electron transfer at the surface and edges.

### Electrochemical and Analytical Measurements

**Figure 2:**
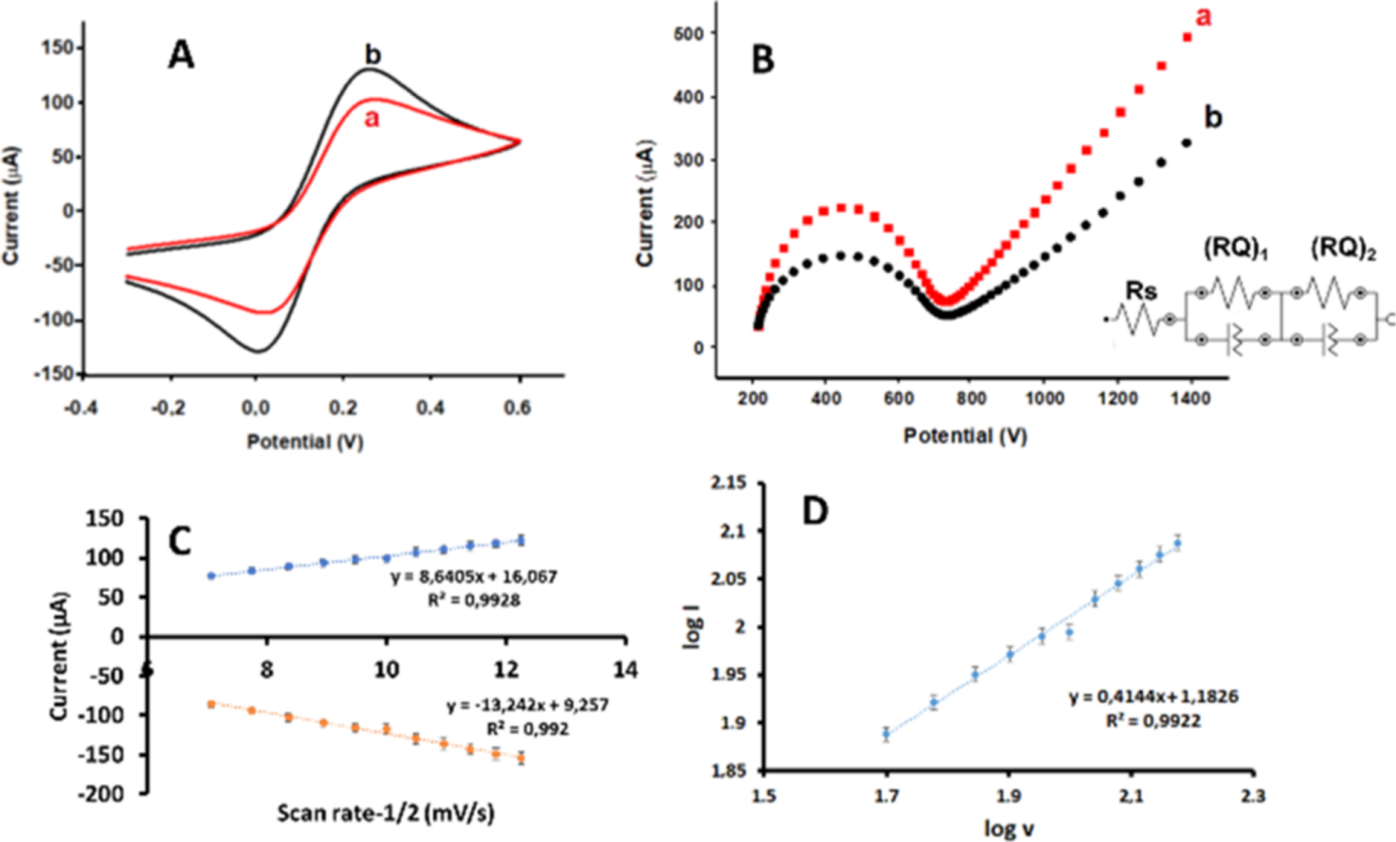
(A) Cyclic voltammetric responses of the (a) bare SPE and (b) CSQD-ZnS/CdSe SPE in 0.1 M KCl and 5 mM Fe(CN)_6_^3/4^ solutions. (B) Nyquist diagrams of the (a) bare SPE and (b) CSQD-ZnS/CdSe SPE in a 0.1 M KCl-containing 5 mM Fe(CN)_6_^3/4^ solution. Inset: equivalence circuit; *R*_s_ refers to the solution resistance, (*RQ*)_1_ and (*RQ*)_2_ refer to the phase layer and diffusion process between solution media and CSQD-ZnS/CdSe SPE layers on the electrode surface, −0.4 +0.6 V, 100 mV s^–1^ scan rate; the frequency range for EIS: 10^–1^ to 10^4^ Hz. (C) Anodic and cathodic peak currents vs scan rate^–1/2^ graphic. (D) Log *I* vs log *v* graphics for the kinetic behavior enlightenment in a 0.1 M KCl-containing 5 mM Fe(CN)_6_^3/4^ solution.

Since the quantum dot family is useful for electrochemical sensing, CSQD-ZnS/CdSe SPEs were chosen for the present study. The electrochemical characteristics of a CSQD-ZnS/CdSe SPE were determined using the cyclic voltammetry (CV) and electrochemical impedance spectroscopy (EIS) methods and compared with those of a bare carbon SPE (Figure 2). The kinetic behavior of the CSQD-ZnS/CdSe SPE will be examined in detail in further sections, but in brief, a general enhancement of the current response was observed with the presence of CSQD-ZnS/CdSe structures on the SPE (Figure 2A). The peak value of the modified SPE (Figure 2A(b)) was significantly higher than that of the bare SPE (Figure 2A(a)).

**Figure 3:**
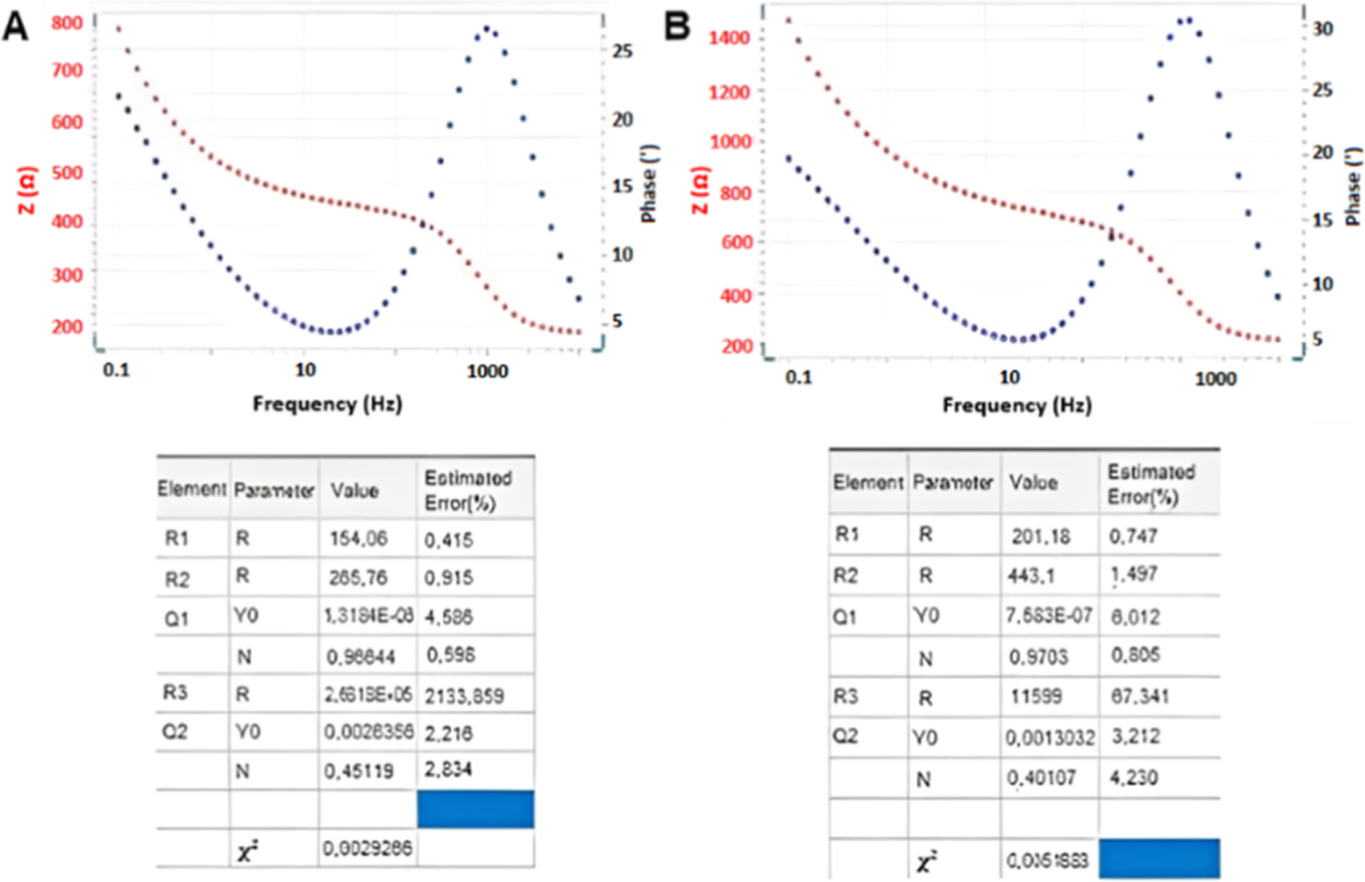
Bode phase diagrams and circuit summary of the (A) bare SPE and (B) CSQD-ZnS/CdSe SPE.

EIS was used to confirm the electrochemical mechanism observations based upon the CV voltammograms.^14^ The Nyquist plots of the bare SPE and CSQD-ZnS/CdSe SPE are illustrated in Figure 2B. According to the fitting analysis, a Randles-type spectrum was defined for the electrodes, including the Warburg phase as a linear part. The fitting analysis of the utilized software showed the goodness of fit with the chi-squared value. The best-fitting circuit was obtained for *R*_s_(*RQ*)_1_(*RQ*)_2_, (*R*_s_: resistance of the electrolyte, *R*: inner resistance, and *Q*: inner capacitance or other capacitive elements) with the lowest estimated errors as 0.003 and 0.005 for the bare SPE and CSQD-ZnS/CdSe SPE, respectively, and the circuit is given as an inset in Figure 2B. The EIS technique helped to understand the impedance changes due to the different interfaces. In this technique, the *R*_ct_ value increases as the charge transfer resistance increases. As the impedance on the surface increases, the *R*_ct_ value seen in the semicircle decreases. Since quantum dots increase conductivity, it is expected that the *R*_ct_ value will be smaller than that of the bare electrode.^15^ In the Nyquist plots, the resistive charge transfer values obtained from the semicircles were 123 and 154 Ω for the bare CSQD-ZnS/CdSe SPE and SPE, respectively. With the presence of CSQD-ZnS/CdSe on the SPE surface, the corresponding electroactive surface is enhanced; the resistive charge transfer value is decreased (Figure 2B) upon increasing the current value, as clearly indicated in Figure 2A. The regression equations for the electrode, according to the graph of current vs square root of the scan rates (Figure 2C), were *y* = 8.6405*x* + 16.067 (*R*^2^ = 0.9928) for the anodic region and *y* = −13.242*x* + 9.257 (*R*^2^ = 0.992) for the cathodic region; the slopes indicated that there was a quasi-reversible process on the electrode surface. In general, the slope value of the log *I* (μA) vs the log *v* graphic defined the diffusion-controlled process at 0.5, and the adsorption-controlled system showed a slope value of 1. The regression equation of the CSQD-ZnS/CdSe SPE was log *I* (μA) = 0.4144 log *v* + 1.1826 (*R*^2^ = 0.9922) (Figure 2D). Since the obtained slope value was 0.4144, the electron transfer mechanism could be defined as diffusion-controlled.^16^ Bode phase diagrams and circuit summaries of the corresponding bare SPE and CSQD-ZnS/CdSe SPE are provided in Figure 3. CV voltammograms of the CSQD-ZnS/CdSe SPE are provided in Figure S1 (Supporting Information).

Increasing scan rates were applied to the electrode, from 50 to 150 mV s^–1^, in 10 mV s^–1^ increments; the current and potential values are given in Table 1.

**Table 1:** Current and Potential Values of CSQD-ZnS/CdSe SPE at Increasing Scan Rates^a^

scan rate (mV s^–1^)	*E*_pa_ (V)	*I*_pa_ (μA)	*E*_pc_ (V)	*I*_pc_ (μA)	Δ*E* (V)
50	0.2250	77.28	0.0395	85.51	0.1855
60	0.2372	83.50	0.0444	93.74	0.1928
70	0.2568	89.26	0.0492	102.82	0.2075
80	0.2665	93.63	0.0517	109.38	0.2148
90	0.2763	97.85	0.0517	115.61	0.2246
100	0.2787	98.80	0.0541	117.35	0.2246
110	0.2885	106.94	0.0492	129.58	0.2392
120	0.2983	110.98	0.0492	136.22	0.2490
130	0.3056	114.96	0.0468	142.56	0.2587
140	0.3105	118.99	0.0444	148.73	0.2661
150	0.3154	122.35	0.0444	153.93	0.2709

a *E*_pa_: anodic peak potential, *E*_pc_: cathodic peak potential, *I*_pc_: cathodic peak current, *I*_pa_ anodic peak current, and Δ*E*: total peak potential.

Here, we monitored the 0.1 M KCl and 5 mM Fe(CN)_6_^3/4^ electrolyte system. The anodic and cathodic peaks corresponded to 0.2250 and 0.0395 V, respectively, at a scan rate of 50 mV s^–1^. The ratio of the peak currents indicates a completely reversible process, and the peak separation value increases proportionally by scan rates. This behavior indicates enhanced diffusion-controlled electron transfer on the electrode’s surface.

**Figure 4:**
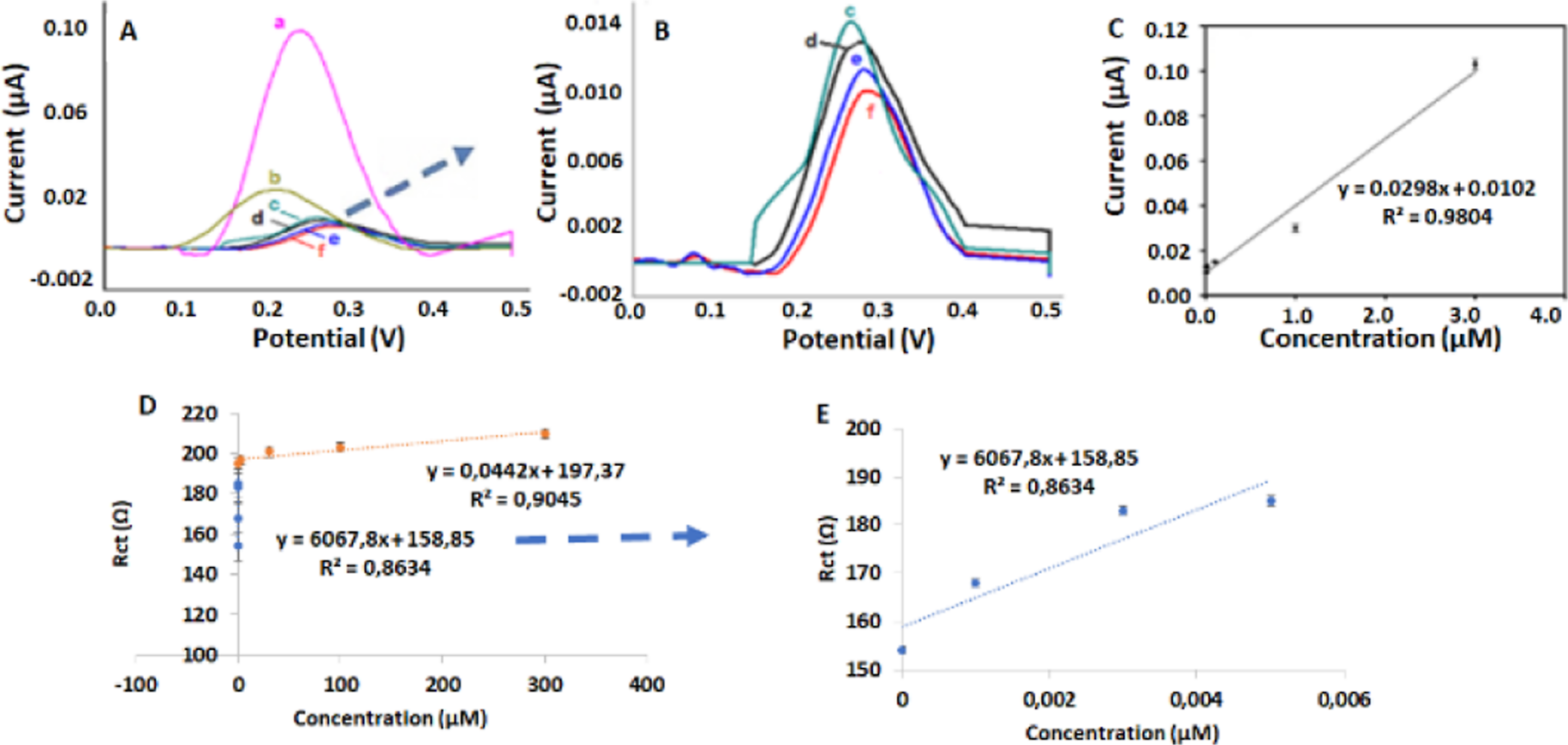
A) Differential pulse voltammetry (DPV) voltammograms of the CSQD-ZnS/CdSe SPE for different concentrations of adrenaline [(a) 3, (b) 1, (c) 0.1, (d) 0.005, (e) 0.003, and (f) 0.001 μM]. (B) Magnification of the lower concentrations. The calibration plots are based upon (C) DPV voltammograms, (D) EIS spectra, and (E) magnification of the lower linear range of adrenaline in phosphate-buffered saline (PBS) with KCl.

The analytical determination of adrenaline in PBS using the CSQD-ZnS/CdSe SPE was successfully examined in detail using DPV (Figure 4A,B; magnified voltammograms given in A and C) and EIS (Figure 4D,E; magnification of the lower linear range graph). The CSQD-ZnS/CdSe SPEs were examined using DPV for varying adrenaline concentrations. The results provided a wide linear concentration range, 0.001 μM to 3 μM, with the equation *y* = 0.0298*x* + 0.0102 (*R*^2^ = 0.9804; Figure 4C). The best limit of detection (LOD) was 2.85 × 10^–8^ μM from the DPV measurements (*n* = 3; Figure 4A–C). The EIS graphics were also evaluated as an alternative method. According to the obtained resistive charge transfer values of the different adrenaline concentrations, additional calibration was obtained. The validated data revealed two linear-ranged calibration plots; the 0.001–0.5 μM region presented the correlation equation *y* = 6067.8*x* + 158.85 (*R*^2^ = 0.8634), and the 0.1–300 μM region showed *y* = 0.0442*x* + 197.37 (*R*^2^ = 0.9045). The results indicated that both the DPV and EIS methods were appropriate for detecting adrenaline concentrations with CSQD-ZnS/CdSe SPEs.

### Measurements of Adrenaline in Solution

**Figure 5:**
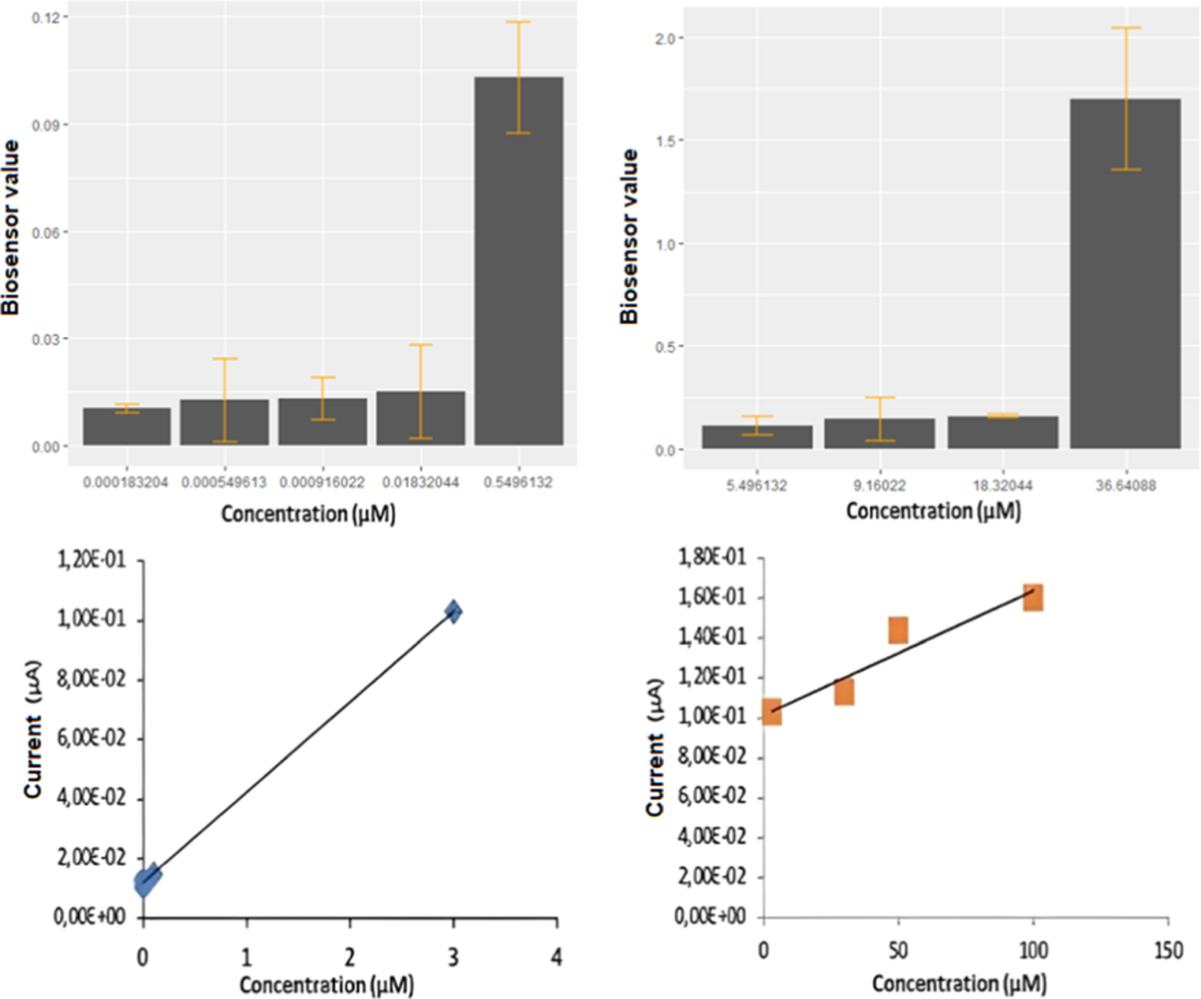
Bar plots and correlation values for the biosensor measurements of adrenaline concentrations from solution.

Figure 5 illustrates the relationship between the adrenaline concentrations obtained from the adrenaline solution prepared in the laboratory and the biosensor values. There was a strong positive correlation between the two measured concentrations and the sensor values (*r* = 0.99; *p* < 0.001).

### Measurements from a Healthy Individual

**Figure 6:**
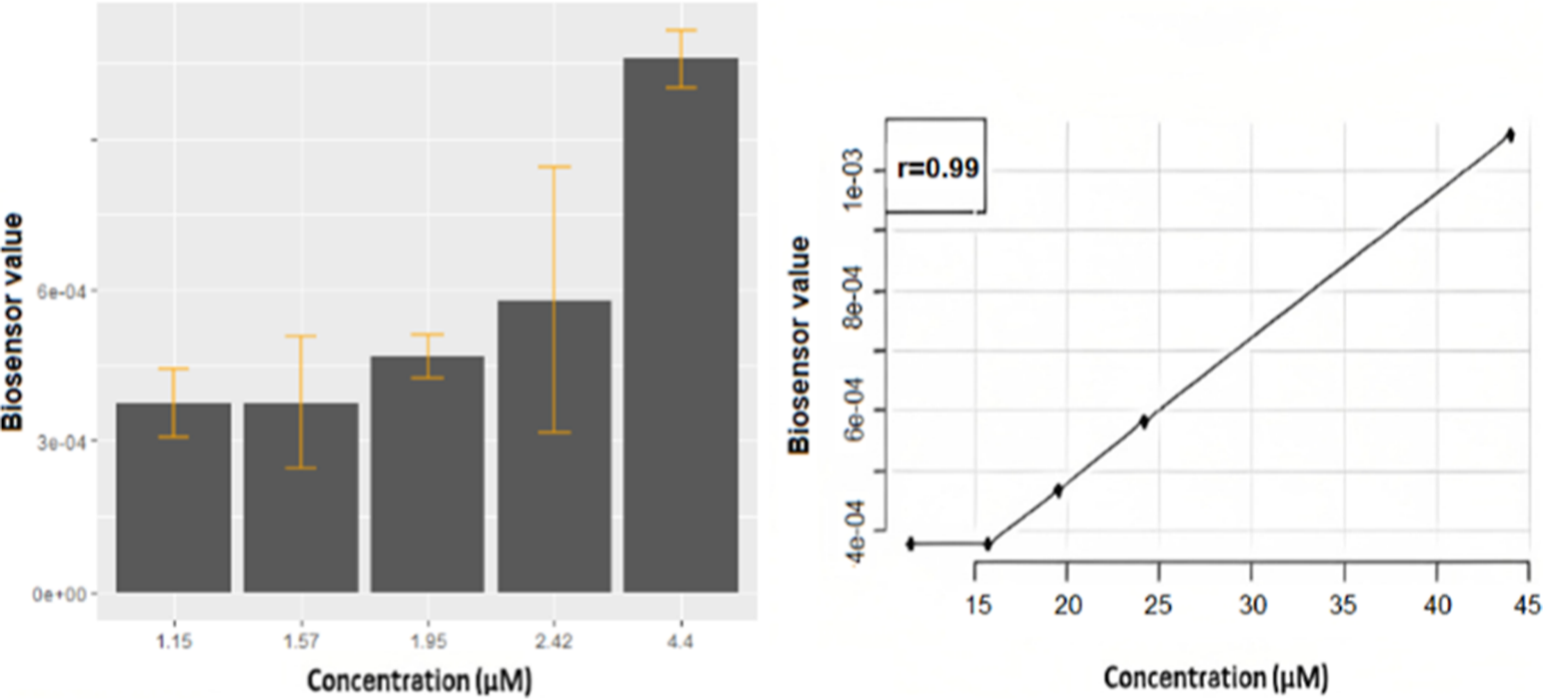
Relationship between the adrenaline values measured from a healthy person and the biosensor values.

Figure 6 illustrates the amount of adrenaline measured from a sample taken from a healthy individual. These graphs show a 99% positive correlation between the biosensor values and the amounts of adrenaline in the blood. This value is statistically significant (*p* < 0.05) and indicates that the sensor can obtain measurements with almost 99% accuracy.

### Measurements in Patients Undergoing CPR

Twenty patients were included in this part of the study. Their median age was 82 (54–94) years, and 45% were women. Spontaneous circulation was not achieved in any of the patients. Demographic data of patients, adrenaline doses administered, and measured adrenaline amounts are provided in Table S1 (Supporting Information).

Three separate measurements were made for each sample in the same conditions. There was no significant difference between the administered adrenaline amount and the measured values in terms of gender for any of the three measurements (Table S2) (Supporting Information).

Figure S2 illustrates the correlations between the adrenaline administered and three sensor-acquired measurements (Supporting Information). A strong positive correlation (*r*_1_ = 0.865; *p* < 0.001) was observed for the first measurement, and moderate positive correlations (*r*_2_ = 0.760, *p* < 0.001; *r*_3_ = 0.586, *p* = 0.007) were observed for the second and third measurements.

In this study, we aimed to develop a biosensor capable of determining exogenous blood adrenaline levels in patients undergoing CPR. The modified electrode used for this purpose showed good reproducibility, stability, and sensitivity and successfully detected the adrenaline levels in these patients.

Adrenaline and its impact on patients have been evaluated predominantly using observational studies rather than randomized trials. Recommendations regarding adrenaline are primarily based on animal data and the associated positive short-term effects and survival to hospital admission.^17,18^ There is uncertainty regarding the amounts and numbers of doses of adrenaline given. One study reported no difference in survival between individuals who received repeated administrations of high-dose (5 mg) and standard-dose (1 mg) adrenaline but reported a slight increase in ROSC in the high-dose group.^19^ Improved short-term survival has been reported in patients receiving adrenaline, while worse long-term survival and functional outcomes have been reported.^20,21^ While a recent study found that adrenaline improved survival at 12-month follow-up, there was no evidence of improvement in favorable neurological outcomes.^22^ In a study examining plasma catecholamine levels before the administration of adrenaline in patients with cardiopulmonary arrest, plasma adrenaline levels were found to be significantly lower in the group with ROSC. Therefore, it was deduced that increased adrenaline levels in plasma may not be associated with ROSC in patients with cardiopulmonary arrest.^23^ A biosensor that can detect adrenaline levels in the blood during CPR can help us discover new information about adrenaline.

Electrochemical methods are preferred because they are simple and fast and do not require any preliminary preparation or expensive equipment. Modifying a bare electrode surface with various materials is an effective method for determining adrenaline levels. In the literature, nanomaterials have been widely used to modify electrode surfaces for the measurement of adrenaline levels.^24−28^ In our study, modified CSQD-ZnS/CdSe SPEs were used. In other studies, examining adrenaline using DPV, LOD values ranged from 0.029 to 0.65 μM.^24,26,27,29^ A superior LOD value (2.79 × 10^–8^ μM) was achieved in our study. Lower LOD values mean that the sensor can detect the analyte at lower concentrations. These results comprise a significant contribution to the measurement of adrenaline levels during CPR. In addition to the modified electrode’s analytical advantages, it is also practical for sensor applications due to its miniaturized structure as an SPE. Comparisons of previous, similar studies are given in Table 2.

**Table 2:** Comparison of the Analytical Performance of CSQD-ZnS/CdSe SPE with Previously Reported Modified Electrodes and Methods for the Detection of Adrenaline^a^

sensor matrix	detection limit	linear range	methods
zeolite-modified carbon paste electrode doped with iron(III)^24^	0.44 μM	0.9–216 μM	DPV
mesoporous SiO_2_-modified carbon paste electrode^25^	0.6 μM	0.1–60 μΜ	CV
ferrocene-modified CNT paste electrode^26^	0.2 μM	0.5–200 μM	DPV
multiwalled CNT-modified carbon paste electrode^27^	0.029 μM	0.03–500 μΜ	DPV
NiO/CNT nanocomposite-modified carbon paste electrode^28^	0.01 μM	0.08–900 μM	SWV
hydroquinone derivative and graphene oxide nanosheet-modified carbon paste electrode^29^	0.65 μM	1.5–600 μM	DPV
niacin film-coated carbon paste electrode^30^	0.011 μM	20.6–174.4 μM	CV
MXene/GCPE^31^	0.009 μM	0.02–10 μM	CV
polyoxalic acid-modified carbon nanotube paste electrode^32^	3.1 × 10^–8^ M	1.0 × 10^–5^, 1.1 × 10^–4^ M	CV
titanium oxide nanoparticle-modified carbon paste electrode^33^	4.2 μM	10 to 100 μM	CV
poly(Allura red)-modified carbon paste electrode^34^	6.8 μM	10 to 80 μM	CV
this work (CSQD-ZnS/CdSe SPE)	2.79 × 10^–8^ μM	0.001–3 μM	DPV

a CV: cyclic voltammetry, DPV: differential pulse voltammetry, SWV: square-wave voltammetry.

There are some limitations to this study. First, the rapid metabolization of adrenaline after administration poses limitations for its measurement. Second, adrenaline is a catecholamine that is also found endogenously in humans. Third, the adrenaline levels of patients with cardiopulmonary arrest were not studied prior to the administration of CPR. Finally, all of the patients included in the study were patients who could not achieve ROSC. Because the aim of this study was to measure the adrenaline levels in blood taken in the middle of resuscitation in patients who underwent CPR, a comparison with ROSC was not considered.

## Conclusions

In the present study, the exogenous adrenaline levels in patients undergoing CPR were determined on site with electrochemical DPV and EIS methods using ultrasensitive ZnS/CdSe-loaded SPE platforms for the first time. A very low LOD (2.79 × 10^–8^) μM was achieved. Two different methods were applied to detect the linear ranges and 0.001–3 μM was achieved from DPV and 0.001–300 μM from the EIS method. Twenty patients were included for the real-time measurements of the study. Their median age was 82 (54–94) years, and 45% were women. A strong positive correlation (*r*_1_ = 0.865; *p* < 0.001) was observed for the first measurement, and moderate positive correlations (*r*_2_ = 0.760, *p* < 0.001; *r*_3_ = 0.586, *p* = 0.007) were observed for the second and third measurements. The modified electrode successfully measured exogenous blood adrenaline levels and showed good reproducibility, stability, and sensitivity.

## Experimental Section

### Study Design

The study was carried out in three stages. First, electrochemical adrenaline measurements were carried out in aqueous adrenaline solutions that were prepared in the different concentration ranges as 0.001–3 μM for DPV and 0.001–300 μM for EIS methods. In the second stage, adrenaline concentrations were measured from plasma samples taken from healthy volunteers. Third, adrenaline concentrations were measured from plasma samples taken from patients undergoing CPR. The demographic and clinical characteristics of the patients who underwent CPR, the amounts of adrenaline administered, and the duration and results of CPR were recorded.

Twenty patients who underwent CPR in the emergency department were included in the study. The blood samples taken from the patients who underwent CPR were collected into tubes with EDTA, centrifuged, and kept at +4 °C for 1 h before undergoing the measurement process. Patients for whom CPR was initiated outside the hospital were not included in the study.

### Ethical Approval

Approval for this study was obtained from the local ethics committee (decision no: 2022/68). Researchers participated in the CPR practice as observers. Informed signed consent was obtained from the relatives of the patients.

### Experimental Section

50 μM PBS was prepared with KH_2_PO_4_, deionized water, and KCl as a supporting electrolyte. The probe solution was 5 mM K_3_Fe[CN]_6_ and K_4_Fe[CN]_6_ including PBS. All of the chemicals were of analytical grade and purchased from Sigma-Aldrich. The electrochemical behavior of electrodes was investigated using CV with a scanning rate of 100 mV s^–1^ between −0.4 and +0.6 V and DPV with a scanning rate of 100 mV s^–1^ between +0.1 and +0.4V, and the frequency range for EIS = 10^–1^ to 10^4^ Hz.

### Apparatus

CV, EIS, and DPV measurements were performed using an AUTOLAB-PGSTAT 204 (Metrohm) device equipped with NOVA 2.1.4 software. The CSQD-ZnS/CdSe SPEs were purchased from Dropsens. A Zeiss Sigma 300 scanning electron microscope was used for imaging.

### Sample Preparation

The samples containing adrenaline in solution were prepared by dilution of the 0.5 mg/mL adrenaline ampoules with 50 μM PBS (pH 7.4). The dilutions were made according to the general *M*_1_*X V*_1_ = *M*_2_*X V*_2_ dilution formula to achieve the final concentrations of adrenaline on the SPE electrode surface at the final volume of 40 μL. Therefore, initially, a two-step dilution was made as a 1:1000:100 ratio to reach a reasonable concentration beginning from 0.5 mg/mL adrenaline ampoule. 50 μM PBS (including 50 mM KH_2_PO_4_, and 0.1 M KCl) and 5 mM K_3_Fe[CN]_6_ in PBS were prepared with KH_2_PO_4_, K_4_[Fe(CN)_6_]·3H_2_O, K_3_Fe[CN]_6_, and KCl. These chemicals and NaOH; 98.00% were purchased from Sigma-Aldrich (https://www.sigmaaldrich.com). All the chemicals were of analytical grade and used as received, without any further purification.

### Statistical Method

R software was used for the statistical analyses. Continuous variables were reported as medians and minima/maxima. Categorical variables were presented as frequencies and percentages. Student *t*-tests for independent samples were used to examine the gender-based differences between the measured values. Pearson correlation analyses were used to examine the relationships between the amounts of adrenaline delivered and the measured adrenaline levels. Results were considered significant at *p* < 0.05..

## Supporting Information Available

Cyclic voltammograms of the CSQD-ZnS/CdSe SPE; correlation plots for the biosensor measurements and the amounts of adrenaline administered; demographic data of patients, adrenaline doses administered, and measured adrenaline amounts; and gender-based comparison of adrenaline values measured by biosensors (PDF)

## Abbreviations

CCR2: CC chemokine receptor 2
CCL2: CC chemokine ligand 2
CCR5: CC chemokine receptor 5
TLC: thin-layer chromatography
