# Supplementary material for: Determination of Exogenous Adrenaline Levels in Patients Undergoing Cardiopulmonary Resuscitation
Source: ACS Omega. 2023 May 25;8(22):19425–32. doi: 10.1021/acsomega.3c00555 (PMC10249028; doi:10.1021/acsomega.3c00555)
Supplement: Supplementary file 1 — ao3c00555_si_001.pdf [file ao3c00555_si_001.pdf]

Determination of exogenous adrenaline levels in patients undergoing cardiopulmonary resuscitation

Mehmet Altuntaş,[1#] Derya Bal Altuntaş,[2#] Sema Aslan,[3#] Ersin Yılmaz,[4#*] Ercan Nalbant,[5#]

[1] : M.D., Recep Tayyip Erdoğan University, Faculty of Medicine, Department of Emergency Medicine, Rize, Turkey

[2] : Ph.D., Recep Tayyip Erdoğan University, Faculty of Engineering and Architecture, Department of Bioengineering, Rize, Turkey

[3] : Ph.D., Department of Chemistry, Faculty of Science, Muğla Sıtkı Koçman University, Muğla, Turkey

[4] : Ph.D. Student, Department of Statistics, Muğla Sıtkı Koçman University, Muğla, Turkey

[5] : M.D., Department of Emergency Medicine, Rize State Hospital, Rize, Turkey

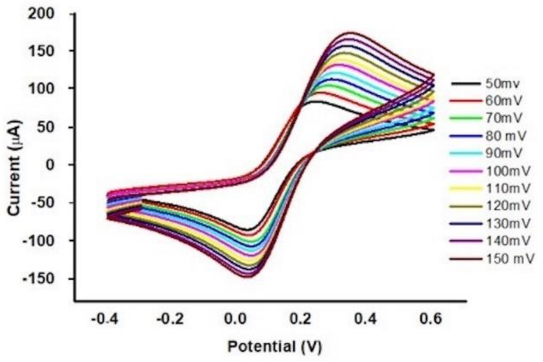

Figure S1: CV voltammograms of the CSQD-ZnS/CdSe SPE.

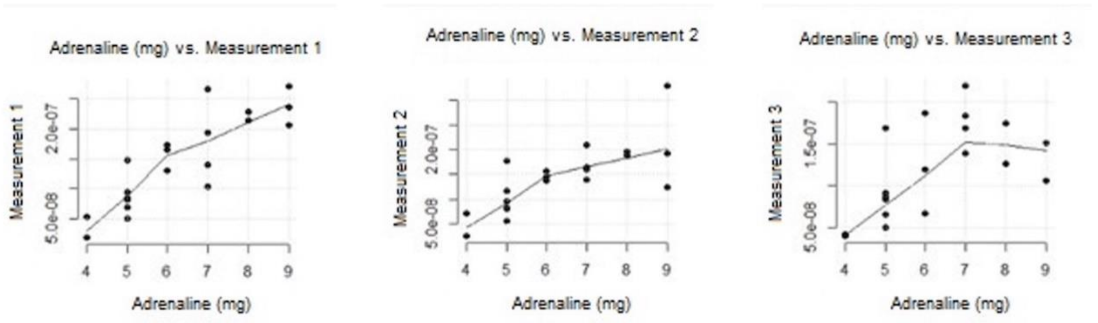

Figure S2: Correlation plots for the biosensor measurements and the amounts of adrenaline administered.

Table S1. Demographic data of patients, adrenaline doses administered, and measured adrenaline amounts

No	Age, G	Dose	M_1	M_2	M_3
**1**	90, M	6	1.65×10^{-7}	1.38×10^{-7}	1.19×10^{-7}
**2**	84, F	5	0.94×10^{-7}	0.55×10^{-7}	0.50×10^{-7}
**3**	80, F	5	0.68×10^{-7}	0.96×10^{-7}	0.86×10^{-7}
**4**	77, M	8	2.28×10^{-7}	1.88×10^{-7}	1.74×10^{-7}
**5**	54, F	6	1.29×10^{-7}	1.44×10^{-7}	0.67×10^{-7}
**6**	88, F	5	0.81×10^{-7}	0.81×10^{-7}	0.84×10^{-7}
**7**	62, M	7	1.39×10^{-7}	1.63×10^{-7}	1.83×10^{-7}
**8**	83, M	6	1.72×10^{-7}	1.56×10^{-7}	1.87×10^{-7}
**9**	75, M	4	0.52×10^{-7}	0.71×10^{-7}	0.34×10^{-7}
**10**	55, M	7	2.67×10^{-7}	1.39×10^{-7}	2.19×10^{-7}
**11**	74, M	5	0.49×10^{-7}	1.15×10^{-7}	0.65×10^{-7}
**12**	94, F	8	2.13×10^{-7}	1.96×10^{-7}	1.26×10^{-7}
**13**	85, M	4	0.18×10^{-7}	0.25×10^{-7}	0.43×10^{-7}
**14**	93, F	5	0.85×10^{-7}	0.81×10^{-7}	0.91×10^{-7}
**15**	83, M	7	1.04×10^{-7}	1.59×10^{-7}	1.68×10^{-7}
**16**	77, M	9	2.37×10^{-7}	3.29×10^{-7}	1.50×10^{-7}
**17**	73, F	9	2.72×10^{-7}	1.25×10^{-7}	1.05×10^{-7}
**18**	83, M	9	2.06×10^{-7}	1.93×10^{-7}	1.51×10^{-7}
**19**	81, F	7	1.93×10^{-7}	2.09×10^{-7}	1.39×10^{-7}
**20**	88, F	5	1.47×10^{-7}	1.77×10^{-7}	1.69×10^{-7}

Dose: *Dose of AD administered (mg/mL),* ***G:*** *Gender,* ***M:*** *Male,* ***F:*** *Female,* ***M (ng/mL)****: Measurement*

Table S2. Gender-based comparison of adrenaline values measured by biosensors

M	Gender	Mean±Std. Dev.	t-statistics	Degrees of freedom	p-value
**M_1**	Female	$(1.43\pm0.70)\times10^{-7}$	0.181	17.981	0.859
	Male	$(1.49\pm0.84)\times10^{-7}$			
**M_2**	Female	$(1.29\pm0.55)\times10^{-7}$	0.780	17.771	0.446
	Male	$(1.53\pm0.76)\times10^{-7}$			
**M_3**	Female	$(1.02\pm0.37)\times10^{-7}$	1.539	16.717	0.143
	Male	$(1.36\pm0.61)\times10^{-7}$			

M (ng/mL)*: Measurement*
